# Biodegradable Alginate-Chitosan Hollow Nanospheres for Codelivery of Doxorubicin and Paclitaxel for the Effect of Human Lung Cancer A549 Cells

**DOI:** 10.1155/2018/4607945

**Published:** 2018-01-28

**Authors:** Liu Tao, Jie Jiang, Yu Gao, Chao Wu, Ying Liu

**Affiliations:** ^1^Nursing College, Jinzhou Medical University, 40 Songpo Road, Linghe, Jinzhou, Liaoning 121000, China; ^2^Pharmacy School, Jinzhou Medical University, 40 Songpo Road, Linghe, Jinzhou, Liaoning 121000, China; ^3^Department of Medical Oncology, First Affiliated Hospital of Jinzhou Medical University, 40 Songpo Road, Linghe, Jinzhou, Liaoning 121001, China

## Abstract

A biodegradable alginate coated chitosan hollow nanosphere (ACHN) was prepared by a hard template method and used for codelivery of doxorubicin (DOX) and paclitaxel (PTX) to investigate the effect on human lung cancer A549 cells. PTX was loaded into the nanometer hollow structure of ACHN through adsorption method. DOX was coated on surface of ACHN through electrostatic interaction. Drug release studies exhibited a sustained-release effect. According to X-ray diffraction patterns (XRD), differential scanning calorimetry (DSC), and Fourier transform infrared spectroscopy (FT-IR) analysis, DOX structure in the loading samples (DOX-PTX-ACHN) was of amorphous state while PTX was microcrystalline. Cytotoxicity experiments showed ACHN was nontoxic as carrier material and the combination of DOX and PTX in DOX-PTX-ACHN exhibited a good inhibiting effect on cell proliferation. Cell uptake experiments demonstrated that DOX-PTX-ACHN accumulated in the cytoplasm. Degradation experiments illustrated that ACHN was a biodegradable material. In summary, these results clearly indicate that ACHN can be utilized as a potential biomaterial to transport multiple drugs to be used in combination therapy.

## 1. Introduction

In recent years, combination therapy has been widely applied during clinical treatment of cancer [1–4]. It can reduce the frequency and dosage of drug administration, relieve drug resistance, decrease side effects, produce synergy, and enhance the effect of any one drug against tumor cells [5]. Many drug combination regimens have been used in clinical trials. However, chemotherapy drugs possess different physiochemical properties, pharmacokinetics and cellular uptake profiles, and so on [6, 7]. These properties make it very difficult to determine the proper dosing and administration of multiple agents at the same time to an individual patient. Therefore, it is necessary to develop a carrier platform for delivering multiple drugs in order to address these problems.

Currently, specific mechanisms of drug delivery have attracted much attention, such as the use of liposomes [8], nanoparticles [9], and micelles to deliver drugs to the patient [10]. However, the compatibility, stability, and toxicity of excipients must also be taken into account. Nanoporous materials were developed as a platform for synergistic codelivery of drugs. Compared with mesoporous nanoparticles, nanospheres with hollow structures have unique advantages [11, 12]. The shell structure can regulate drug release. In drugs that have poor solubility in water, the nanoscale hollow structure decreases drug particle size and inhibits its crystallinity leading to improved water solubility. Compared with inorganic hollow nanospheres [13–16] (such as hollow silicon nanospheres and hollow carbon nanospheres), organic hollow nanospheres have more advantages, because of the absence of problems regarding degradation and toxicity. The use of chitosan (CS), hyaluronate, sodium alginate, and polylactic acid as frame materials for hollow nanospheres has been widely studied [17–19].

In this study, we designed an alginate coated chitosan hollow nanosphere (ACHN) to serve as a carrier for codelivering paclitaxel (PTX) and doxorubicin (DOX). It is well known that combined administration of PTX and DOX is commonly used in the cancer treatment. PTX was loaded in nanoscale hollow structure by an adsorption method. The positively charged DOX was coated onto the surface of negatively charged ACHN using electrostatic adsorption. Using in vitro drug release experiments, drug morphology characterization in the carrier and cell biology tests, will permit us to determine if ACHN has potential to be used as a carrier molecule for combined therapy in the treatment of lung cancer.

## 2. Materials and Methods

### 2.1. Chemicals and Materials

Paclitaxel was provided by Tianfeng Biotechnology Company with purity > 99%. Doxorubicin was obtained from Hefei Biological Technology Co., Ltd. with purity > 98%. Chitosan (CS) and sodium alginate were purchased from Sinopharm Chemical Reagent Co., Ltd. (Shanghai, China). Sulfuric acid (98%), Tween 80, acetic acid, anhydrous ethanol, tetrahydrofuran (THF), glutaraldehyde (50% water solution), potassium persulfate (KPS), methanol, acetonitrile, hypromellose, dimethyl sulfoxide (DMSO), 2,2′-azobis[2-methylpropionamidine] dihydrochloride (AIBA), dichloromethane, and styrene were provided from Tianjin Yongsheng Fine Chemical Co., Ltd. (Tianjin, China). Annexin V-FITC, propidium iodide (PI), 3-(4,5- dimethylthiazol-2-yl)-2,5-diphenyltetrazolium (MTT), RPMI 1640 medium, trypsin, fetal bovine serum, Tris buffered saline tween solution (TBST), albumin from bovine serum (BSA), phosphate buffered saline (PBS), Hoechst 33342, and rhodamine-phalloidin were purchased from Beijing Dingguo Changsheng Biotech Co., Ltd. (Beijing, China).

### 2.2. Preparation of ACHN

#### 2.2.1. Synthesis of Polystyrene Nanosphere

A soap-free emulsion polymerization method was used to synthesize the monodispersed polystyrene nanospheres (PS) [20]. Briefly, styrene (40 g) was dripped slowly in distilled water (390 ml) containing 1 g DAC (80 wt%). The system was stirred at room temperature for 30 min. Then, nitrogen was blown in the solution for purging oxygen and the solution was heated to 90°C via an oil bath. 10 mL of AIBA solution (0.1 g/ml) was dripped slowly and the system formed emulsion. It was stirred for 24 h at 90°C, and then the products were centrifuged at 10,000 rpm for 30 min. After washing with ethanol three times, the PS was dried at 50°C and stored for the next step.

#### 2.2.2. The Sulfonation of PS

As previously done [21, 22], PS and sulfuric acid (98%) were mixed at a mass ratio of 1 : 40 under ultrasonic conditions. The reaction was carried out for 12 h at 40°C under stirring. The products were centrifuged at 8,000 rpm and washed repeatedly with water in order to remove the sulfuric acid. The obtained sulfonated PS (SPS) was dried at 50°C.

#### 2.2.3. Synthetic Processing of ACHN

SPS template and deionized water were mixed at a mass ratio of 1 : 100 under ultrasonic conditions. 0.01 g/ml of CS solution containing 2% acetic acid was added to the SPS suspension while stirring. After 2 h, the product was obtained by centrifugation and was dispersed again in a sodium alginate solution (1 mg/ml) for 2 h. After centrifugation, the obtained product was dispersed slowly into 5 ml glutaraldehyde (2.5%) under stirring. The reaction was interrupted after 2 h. Then, THF was added to remove the SPS template, and the obtained ACHN was washed repeatedly with ethanol and dried at 50°C.

### 2.3. Drug Loading

The process of loading ACHN with PTX and DOX was as follows. Briefly, 60 mg of ACHN was added to a PTX dichloromethane solution (60 mg/ml) for 6 h under stirring to reach adsorption equilibrium. The loading sample obtained by centrifugation was then added to 50 ml of a polyacrylamide aqueous solution (1 mg/ml) with magnetic stirring for 6 h. After centrifugation, the supernatant was removed. Subsequently, DOX solution (10 mg/ml) was added and stirred for 6 h. The obtained ACHN with PTX and DOX (DOX-PTX-ACHN) was isolated through centrifugation and was dried under vacuum conditions. In order to analyze two drug states in DOX-PTX-ACHN, ACHN only loaded PTX (PTX-ACHN) or DOX (DOX -ACHN) was prepared for comparison.

The amount of PTX in DOX-PTX-ACHN was determined by high-performance liquid chromatography (HPLC) (L-2400, HITACHI, Japan) using a Welch C18 column (4.6 mm × 200 mm, 5 *μ*m) at 227 nm. UV spectrophotometer (UV-2000, Unico, USA) was used to determine DOX content at 490 nm.

### 2.4. Characterization

Transmission electron microscopy (TEM) (Tecnai G2F30, USA) and scanning electron microscopy (SEM) (JEOL JSM-7001F) were used to identify the morphological and structural characteristics of SPS and ACHN. Differential scanning calorimetry (DSC) (DSC-60, Shimadzu, Japan) and X-ray diffraction patterns (XRD) (Rigaku Geigerflex XRD, Co., Japan) were used to characterize the physical properties of PTX and DOX within DOX-PTX-ACHN. Fourier transform infrared spectroscopy (FT-IR) (Bruker IFS 55, Switzerland) was used to determine whether PTX, DOX, and ACHN had an interaction.

### 2.5. Drug Release Studies

In vitro drug release behavior of DOX-PTX-ACHN was performed using a dissolution instrument (ZRS-8G, Tianjin Xintianguang, China). Release conditions were as follows: the temperature was 37 ± 0.5°C, the paddle speed was 100 ± 1 rpm, the release medium was 1000 ml of phosphate buffered solution (PBS, pH 6.8) containing 0.1% SDS, and the drug specification of DOX-PTX-ACHN was 30 mg. The free PTX was used as control. At predetermined time points (1, 2, 4, 6, 8, 12, 24, 36, 48, and 72 h), 5 ml of release medium was withdrawn and blank release medium at the same volume was supplemented. The PTX of the filtered samples at each time point was analyzed by HPLC (HITACHI L-2400, Japan) with a Welch C18 column (200 mm × 4.6 mm, 5 *μ*m). The flow rate was 1 ml/min and the mobile phase was acetonitrile and water with a volume ratio of 50 : 50. The DOX of the filtered samples at each time point was determined by UV spectrophotometer (UV-2000, Unico, USA). The detection wavelength of PTX and DOX was 227 nm and 490 nm, respectively.

### 2.6. Cytotoxicity Assay

A549 lung cancer cells at a density of 1 × 10^5^ cells/well were cultured in 96-well plates under CO_2_ conditions at 37°C. RPMI 1640 medium with 10% fetal bovine serum (FBS) was used as culture medium. The toxicity of ACHN at different concentrations was evaluated by MTT experiments [23]. Briefly, 100 *μ*l of ACHN suspension at different concentrations (10, 50, 100, 250, and 500 *μ*g/ml) was added to each well and incubated for 24 h. Following incubation, 5 mg/ml of MTT solution (20 *μ*l) was added to each well. After incubation for 4 h, 200 *μ*l of DMSO was added to each well to replace the culture medium and shaken for 10 min. Finally, the absorbance values (AS) were read at 492 nm by a microplate reader.

The combined effect of DOX and PTX was analyzed using a combination index (CI). The cell viability assay of free PTX, free DOX, and DOX/PTX combinations at various molar ratios (DOX-PTX) was done as shown previously [24]. The dispersion medium of the samples was DMSO. The combination index (CI_50_) and dose-reduction index (DRI) of different compositions were calculated using the following equation: DRI = IC_X_/D; CI_50_ = 1/DRI_A_ + 1/DRI_B_ = D_A_/IC_50A_ + D_B_/IC_50B_, where the IC_50A_ and IC_50B_ are the drug concentrations of free DOX and free PTX, respectively, used alone to achieve a growth inhibition rate of 50%. D_A_ and D_B_ are the drug concentration of DOX and PTX, respectively, to reach a growth inhibition rate of 50% in combination therapy.

In vitro cell inhibition experiments of DOX-PTX-ACHN were done the same as above. The DOX, PTX, DOX-PTX-ACHN, and DOX-PTX at equal molar ratios were dispersed, respectively, into a 2% hypromellose solution to prepare a suspension with different concentrations (corresponding to 0.005, 0.01, 0.05, 0.1, 0.5, 1, and 5 *μ*g/ml of PTX). The cell inhibitory rate (CIR) was calculated as follows: CIR% = 100%  − AS(test)/AS(control) × 100%. IC_50_ (half maximal inhibitory concentration) [25] was calculated using SPSS software.

### 2.7. Cell Apoptosis Assays

Annexin V-FITC apoptosis detection kit (Beijing Dingguo Changsheng Biotech Co., Ltd.) was applied to detect cell apoptosis. A549 cells at a density of 1 × 10^5^ cells/well were cultured in 6-well plates under CO_2_ conditions at 37°C. DOX-PTX-ACHN suspensions (equivalent to 10 ng/ml of PTX; concentration ratio of DOX and PTX was consistent with drug loading) and DOX-PTX suspensions with same drug ratio were added to each well and incubated for 48 h at 37°C. After removing medium, trypsin was added to each well to digest the cells. Cells were collected following centrifugation and were dispersed in 500 *μ*l binding buffer, followed by the addition of annexin V-FITC (5 *μ*l) and propidium iodide (PI, 5 *μ*l). Following incubation for 10 min, FACSCalibur flow cytometer (Becton Dickinson, Zürich, Switzerland) was used to detect apoptosis of A549 cells.

### 2.8. Western Blot Analysis

Following treatment of A549 cells with either the DOX-PTX-ACHN suspension or the DOX-PTX suspension, an ultrasonic grinding method was used to extract total protein from A549 cells. The concentration of total protein obtained was determined using the BCA method [26]. A solution of the protein samples and buffer at a ratio of 4 : 1 was boiled for 5 min. The mixed solution (10 *μ*l) and the standard protein were loaded onto an SDS-PAGE gel for electrophoresis. The separated proteins were transferred onto a polyvinylidene fluoride (PVDF) membrane. The membrane was blocked using TBST containing 5% skim milk. Following blocking, the primary antibodies (Bax, Bcl-2, and caspase-3) were incubated with the PVDF membrane overnight. Following incubation with the primary antibody, the membranes were incubated with secondary antibody for at least 2 h and stained with enhanced chemiluminescence reagents. The expression level of the proteins was analyzed using a UVP gel analysis system (UVP, LLC, Upland, CA, USA).

### 2.9. Cellular Uptake Study

The amino group of chitosan can be combined with the thiocarbamide group of FITC, allowing ACHN to be labeled with FITC [27]. ACHN was immersed in 1 mg/ml of an FITC-ethanol solution for 5 h; ACHN labeled with FITC (FITC-ACHN) was isolated by centrifugation and dried under vacuum conditions. A549 cells were grown in 6-well plates at a density of 1 × 10^5^ cells/well. FITC-ACHN (25 *μ*g/ml) was added to each well and incubated for 1, 2, and 4 h. After removing the medium, the cells were washed three times with a PBS solution and cells were fixed for 10 min using a 4% paraformaldehyde solution. Following fixation, cells were washed three times with PBS; 1 ml of Triton X-100 (0.1%) was added and incubated for 10 min. Triton X-100 was removed and cells were washed three times with PBS and then incubated with 1% bovine serum albumin solution (BSA) (1 ml) at 37°C for 30 min. The BSA solution was removed and the cells were stained, respectively, with by Hoechst 33342 (1 *μ*g/ml) and rhodamine-phalloidin (1 *μ*g/ml) after being washed three times with PBS. Confocal microscopy (CLSM) was used to observe cellular uptake [28].

### 2.10. Degradation of ACHN

The biodegradability of ACHN was observed using SEM. Briefly, 10 mg of ACHN was added, respectively, to 5 ml of acetate buffer solution (pH 4.5) under shaking at 37°C. At 1, 3, 6, and 12 h, the supernatant was removed by centrifugation, and the degraded ACHN was solidified for 30 min in anhydrous ethanol. Finally, the samples were dried under vacuum conditions and the change of ACHN morphological structure was characterized by SEM.

## 3. Results and Discussion

### 3.1. Morphological Structure and Drug-Loaded Study of ACHN

**Scheme 1:**
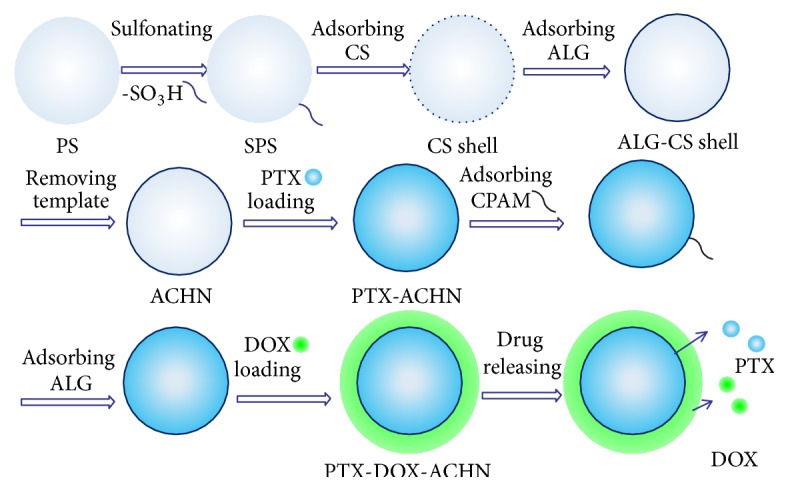
Schematic illustration of preparation of ACHN, the loading of DOX and PTX, and the release from DOX-PTX-ACHN.

**Figure 1:**
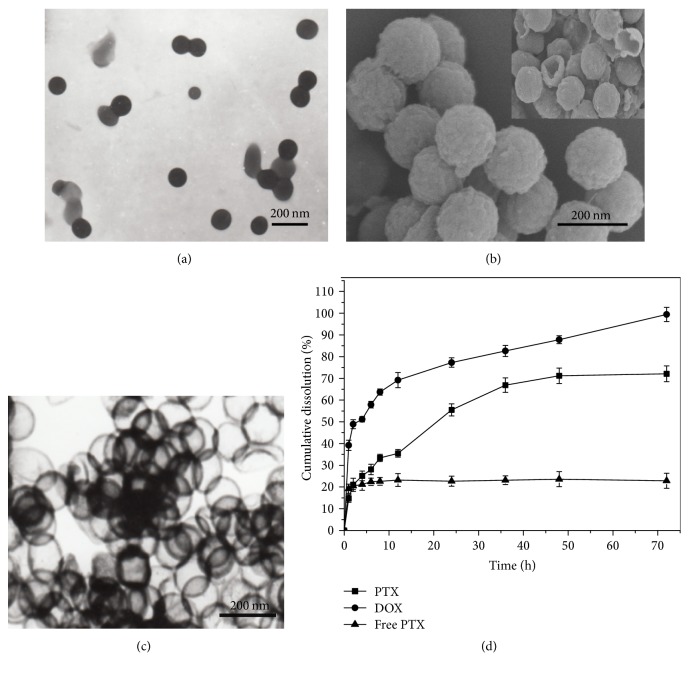
(a) The TEM image of SPS template; (b) SEM image of ACHN; (c) TEM image of ACHN; (d) the drug release curves of free PTX and DOX-PTX-ACHN in PBS (pH 6.8) containing 0.001% SDS at a temperature of 37 ± 0.5°C. Data represented as mean ± SD (*n* = 3).

The formation and drug loading process of ACHN is illustrated in Scheme 1. The hard template method for preparing hollow nanosphere has some advantages such as an adjustable particle size, a simple preparation process, and good monodispersion. The size of PS prepared by emulsion polymerization can be adjusted by changing the amount of initiator (AIBA) and emulsifier (DAC), so it also allows for an adjustable hollow diameter in ACHN. The surface of PS has a negative charge by sulfonation treatment. The positively charged CS molecules are adsorbed on the surface of SPS through electrostatic action. Deposition of CS molecules on the SPS surface forms the core-shell structure of chitosan-SPS (CS-SPS). Negatively charged alginate adsorbs on the positively charged surface of CS-SPS, obtaining the core-shell structure of alginate-chitosan-SPS (Alg-CS-SPS) [29]. PS in Alg-CS-SPS is removed using THF in order to obtain negatively charged ACHN. As shown in Figures 1(b) and 1(c), SEM and TEM images indicated that ACHN had a uniform diameter (about 150 nm) and good monodispersion. The wall thickness of ACHN was about 20 nm. The TEM image in Figure 1(c) presented hollow structure of about 130 nm diameter, which was consistent with the SPS template size (130 nm) in Figure 1(a). The nanoscale hollow structure provides enough space for loading water-insoluble drugs and is suitable for drug storage. The spatial restriction effects of the nanoscale hollow structure could inhibit drug crystallization, reduce drug particle size, and enhance the drug solubility according to the Noyes–Whitney equation and the Ostwald–Freundlich equation. The shell structure plays a role in adjusting the drug release.

To achieve a drug combination, we loaded the hollow structure of the ACHN with PTX using an adsorption method and then coated the negatively charged surface of ACHN with positively charged DOX. Increasing DOX drug loading, the system containing PTX coated with strong cation (polyacrylamide) can adsorb more alginate, which could enhance the negative charge content. The drug loading of PTX and DOX in DOX-PTX-ACHN was 18.4 ± 1.32% and 74.2 ± 3.24%, respectively, and the mole ratio of PTX and DOX in DOX-PTX-ACHN was 1 : 7. Furthermore, as seen in Figure 1(d), drug release results showed that, at 1, 2, 4, 6, 8, 12, 24, 36, 48, and 72 h, the mole ratio of the release amount of PTX and DOX from DOX-PTX-ACHN was less than 1 : 4 and the release process exhibited sustained-release behavior compared with free PTX. The combined effects of DOX and PTX were evaluated by determining the IC50, DRI, and CI50. All parameters are given in Table 1. When the mole ratio of DOX/PTX was more than 1 : 1, the combination had a synergistic effect. In contrast, it showed antagonistic effect for 1 : 4 proportion. DOX and PTX with a mass ratio of 7 : 1 in DOX-PTX-ACHN exhibited an excellent synergistic effect, which is in agreement with previous reports [24, 30].

### 3.2. Drug Morphological Characterization

**Figure 2:**
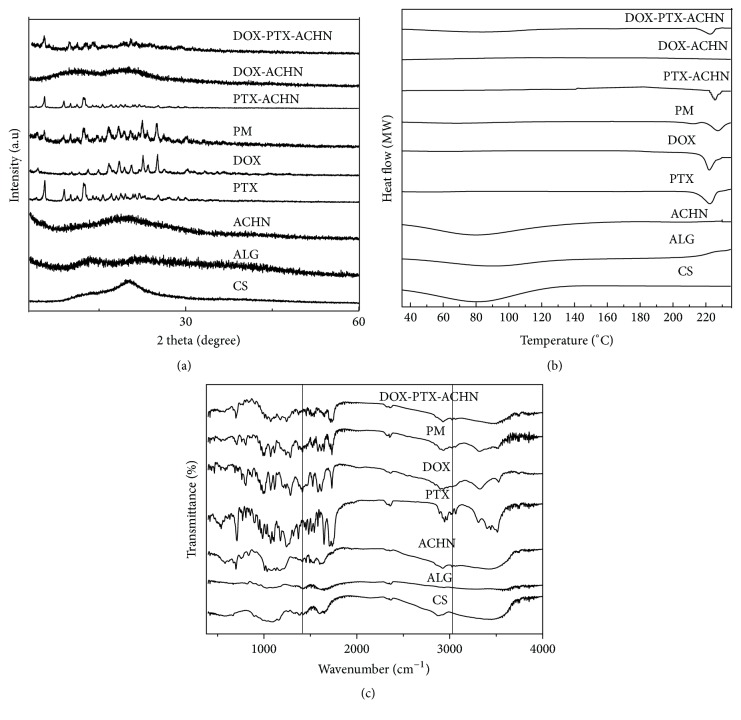
DOX-PTX-ACHN characterization: (a) XRD patterns; (b) DSC patterns; (c) FT-IR patterns.

The results of XRD and DSC demonstrated that PTX was in a microcrystalline state and DOX existed in an amorphous state. As shown in Figure 2(a), XRD patterns of pure PTX and pure DOX displayed many crystalline diffraction peaks and the characteristic diffraction peaks of PTX and DOX at 16.6° and 25.0°, respectively. Compared with the same proportion physical mixture (PM), PTX-ACHN showed a markedly weakened crystalline diffraction pattern, which indicated that the PTX in PTX-ACHN was in a microcrystalline state. DOX-ACHN did not display any crystalline diffraction pattern in comparison with the same proportion physical mixture (PM) and pure DOX, which indicated that DOX was in an amorphous form. XRD patterns of DOX-PTX-ACHN only exhibited weakened crystalline diffraction pattern of PTX and did not have any crystalline diffraction pattern of DOX, which were consistent with the above analysis. As shown in Figure 2(b), DSC patterns showed that the melting points of pure PTX and pure DOX were 222.5°C and 222.1°C, respectively, which illustrated significant overlap of melting temperatures for the two drugs. The endothermic peak of PTX was reduced in PTX-ACHN compared to pure PTX and PM with the same proportion. DOX-PTX-ACHN also had a weakened endothermic peak at 222°C. This demonstrated that PTX in the carrier is in a microcrystalline state. DOX-ACHN did not display any crystalline diffraction pattern, indicating that DOX in DOX-PTX-ACHN was in an amorphous form. The conclusions of XRD and DSC characterization were consistent. A FT-IR spectrum was used to analyze the interaction between ACHN and the two drugs. As shown in Figure 2(c), the FT-IR patterns of DOX-PTX-ACHN were in agreement with the same proportion physical mixture (PM) compared with ACHN, pure PTX, and pure DOX. This indicates that DOX, PTX, and ACHN do not undergo any chemical reactions, and therefore drug adsorption was physical.

### 3.3. Cell Viability

**Figure 3:**
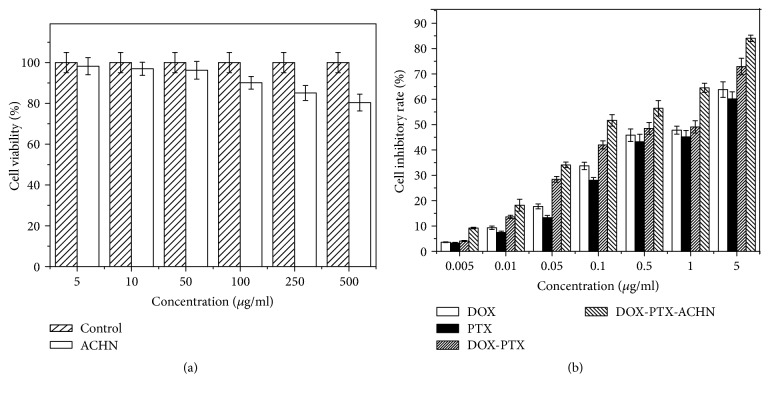
(a) Cell viability of ACHN at different concentrations; (b) cell inhibition of DOX, PTX, DOX : PTX = 7 : 1 (m/m), and DOX-PTX-ACHN at different concentrations. Data represented as mean ± SD (*n* = 6).

The cell viability results illustrated that ACHN was nontoxic and DOX-PTX-ACHN exhibited good synergistic effect on inhibiting A549 cell proliferation. As shown in Figure 3, the cell viability of ACHN at concentrations ranging from 5 *μ*g/ml to 500 *μ*g/ml was more than 80%. This fully demonstrated that ACHN had good biological safety. DOX-PTX-ACHN showed a higher inhibition rate than PTX-DOX at same molar ratio, PTX and DOX. There are two reasons for this result, the first is that the drug combination produced synergistic effects, leading to enhanced effects when compared to a single drug. The second was that the nanosize effect of ACHN could enhance drug absorption, which exposed cells to high concentrations of the drugs thus strongly inhibiting cell proliferation [31].

### 3.4. Apoptosis Analysis

**Figure 4:**
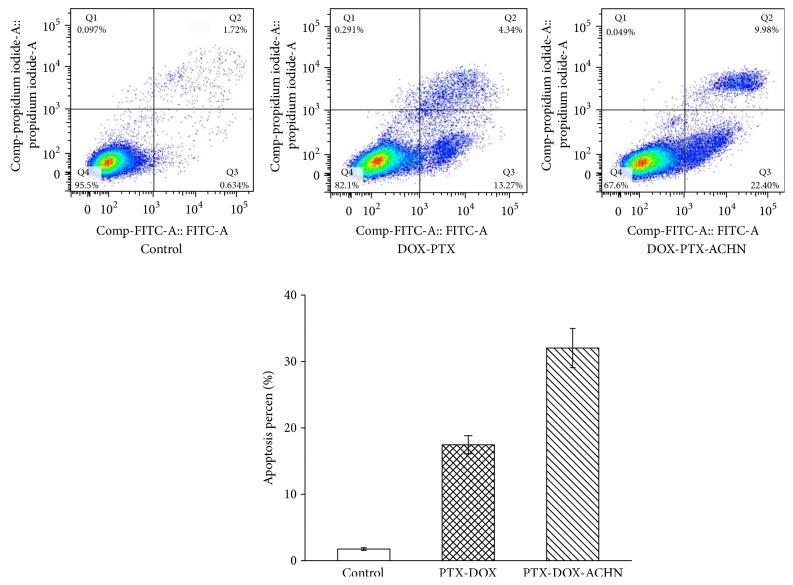
Induction of apoptosis on A549 cells by treatment with DOX-PTX and DOX-PTX-ACHN.

As shown in Figure 4, DOX-PTX-ACHN induced 22.6 ± 2.32% of A549 cell early apoptosis compared to 13.2 ± 1.04% of DOX-PTX with same proportion, indicating DOX-PTX-ACHN had a good effect in promoting apoptosis of A549 cells. The reason for these results was that the combination of PTX and DOX produced synergistic effects and that the accumulation effect of DOX-PTX-ACHN in A549 cells increased the PTX and DOX concentration. The two factors worked together to promote cell apoptosis.

**Figure 5:**
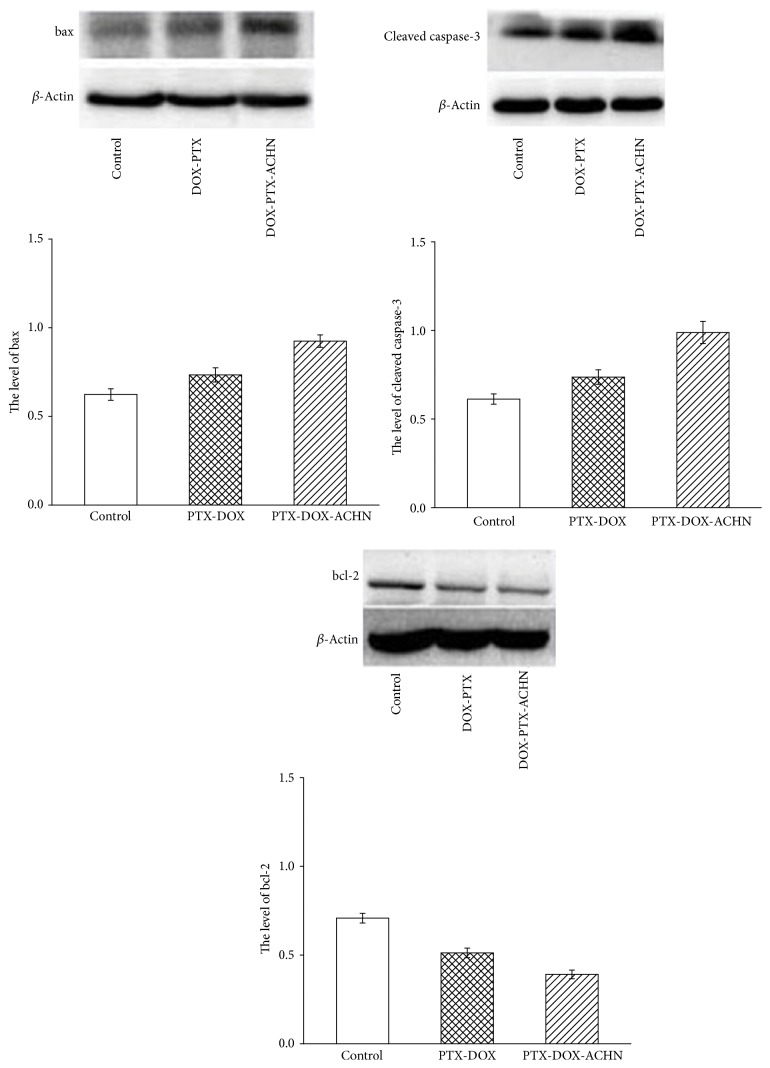
Apoptotic effects of DOX-PTX and DOX-PTX-ACHN on A549 cells by Western blot analysis of the expression levels of Bax and Bcl-2 and cleaved caspase-3 proteins after treatments.

Apoptosis, also known as programmed cell death (PCD), is an active and orderly process of self-destruction under certain physiological or pathological conditions. Bcl-2 protein, caspase protein, and Bax protein have attracted much attention during the process of cell apoptosis. The proapoptotic protein Bax in the cytoplasm can shift by oligomerization to the mitochondrial membrane, promote permeabilization, and release various apoptotic factors which activate the cascade reaction of caspase protein. Finally, activated caspase-3 promotes cell apoptosis. The antiapoptotic protein Bcl-2 on the outer membrane of the mitochondrion can combine with the proapoptotic protein Bax to form heterodimers, which prevents the oligomerization and shift of the apoptosis protein and inhibits cell apoptosis [32]. The protein expression levels of Bax, Bcl-2, and caspase-3 were shown in Figure 5. Compared with the control and free DOX-PTX at the same molar ratio, DOX-PTX-ACHN had less protein expression of Bcl-2 in A549 cells, while the expression of key apoptosis proteins Bax and caspase-3 was significantly increased. The data showed that DOX-PTX-ACHN significantly inhibited protein expression of Bcl-2 in A549 cells and promoted protein expression of Bax and the cleaved caspase-3, which meant that DOX-PTX-ACHN was better at inducing apoptosis of A549 cells. The codelivery system of PTX and DOX by ACHN could effectively prompt the apoptosis of A549 cells to achieve antitumor activity. The above conclusions were consistent with the MTT results.

### 3.5. Cell Uptake Study

**Figure 6:**
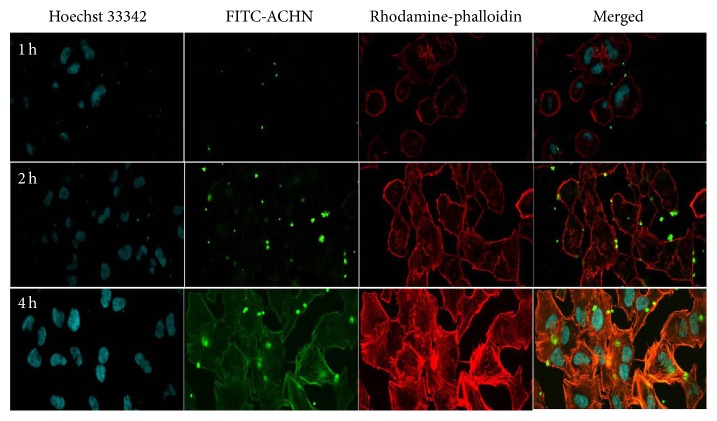
CLSM images of A549 cells treated with FITC-ACHN incubated for 1 h, 2 h, and 4 h.

Cell uptake results demonstrated that FITC- ACHN can be effectively taken into A549 cells.  Figure 6 showed the CLSM images of A549 cells treated with FITC- ACHN at 1, 2, and 4 h. According to the changes of green fluorescence in A549 cells, FITC- ACHN was delivered into the cytoplasm via the cell membrane at 1 h, and the cellular uptake into A549 cells increased proportionately with time, indicating a time dependence.

### 3.6. Degradation Experiments

**Figure 7:**
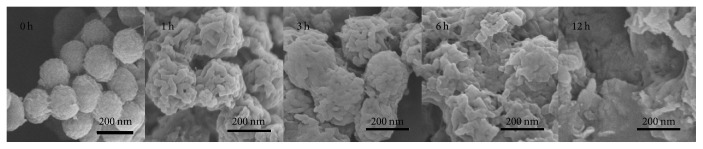
Degradation images of ACHN in an acetate buffer solution (pH 4.5) at different time points.

It is generally known that the pH of the microenvironment in tumor cells is acidic (pH 3–5), which means that ACHN should be degraded in an acidic environment if it was to serve as a drug carrier. As seen in Figure 7, ACHN gradually degraded with increase of time in an acetate buffer solution (pH 4.5). At 1 h, the surface became rough. When the degraded time reached 12 h, ACHN lost its spherical shape and the hollow structure collapsed. These results indicated that ACHN was pH sensitive. This suggests that when DOX-PTX-ACHN accumulates in the cytoplasm of A549 cells, degradation occurs, leading to drug release and enhanced drug concentrations in the tumor, resulting in tumor cellular death.

## 4. Conclusions

ACHN can be loaded successfully with DOX and PTX with the aid of electrostatic and physical adsorption. PTX and DOX in DOX-PTX-ACHN present at an amorphous state and at a microcrystalline state, respectively. The in vitro cell experiment results show that the codelivery system of DOX-PTX-ACHN can effectively inhibit cell proliferation and promote cell apoptosis due to the nanoscale effect of ACHN and the synergistic effect of combined administration of DOX and PTX. From the above findings, biodegradable ACHN is a promising carrier when utilizing drug combinations to enhance antitumor efficacy.

## Figures and Tables

**Table 1:** CI50 of different treatment compositions of drugs to A549 cells after 48 h incubation.

	IC50 (ng/ml)	CI50	DRI-DOX	DRI-PTX
DOX	422.34	/	/	/
DOX/PTX = 7/1	106.24/15.36	0.58	3.98	3.01
DOX/PTX = 4/1	76.13/19.38	0.60	5.55	2.39
DOX/PTX = 1/1	61.17/60.49	1.45	6.90	0.76
DOX/PTX = 1/4	54.16/215.42	4.78	7.80	0.21
PTX	46.26	/	/	/
